# The effect of regular dental cast artifacts on the 3D superimposition of serial digital maxillary dental models

**DOI:** 10.1038/s41598-019-46887-1

**Published:** 2019-07-19

**Authors:** Eva Henninger, Georgios Vasilakos, Demetrios Halazonetis, Nikolaos Gkantidis

**Affiliations:** 1 Department of Orthodontics and Dentofacial Orthopedics, University of Bern, Freiburgstrasse 7, CH-3010 Bern, Switzerland; 2 Private practice, Frankfurter Strasse 610, 51145 Cologne, Germany; 3 Department of Orthodontics, School of Dentistry, National and Kapodistrian University of Athens, 2 Thivon Street, Goudi, 11527 Athens, Greece

**Keywords:** Oral anatomy, Outcomes research

## Abstract

Superimpositions of serial 3D dental surface models comprise a powerful tool to assess morphological changes due to growth, treatment, or pathology. In this study, we evaluated the effect of artifacts on the superimposition outcome, using standard model acquisition and superimposition techniques. Ten pre- and post-orthodontic treatment plaster models were scanned with an intraoral scanner and superimposed using the iterative closest point algorithm. We repeated the whole process after manual removal of plaster artifacts, according to the current practice, as well as after re-scanning the cleaned models, to assess the effect of the model acquisition process derived artifacts on the superimposition outcome. Non-parametric multivariate models showed no mean effect on accuracy and precision by software settings, cleaning status (artifact removal), or time point. The choice of the superimposition reference area was the only factor that affected the measurements. However, assessment of individual cases revealed significant differences on the detected tooth movement, depending on artifact removal and on the model acquisition process. The effects of all factors tended to decrease with an increase in the size of the superimposition reference area. The present findings highlight the importance of accurate, artifact-free models, for valid assessment of morphological changes through serial 3D model superimpositions.

## Introduction

Orthodontic diagnosis and treatment planning is traditionally done with the aid of plaster dental models. Over the past years, three-dimensional (3D) imaging technologies have been highly evolved and are currently incorporated in everyday practice, offering increasing processing capabilities compared to the plaster dental models^1,2^. Additional advantages are that digital dental models are not prone to physical destruction and do not require physical storage.

Nowadays, 3D digital dental models are produced through direct 3D intraoral scanning or through scanning of plaster dental models. Intraoral scanning is an easy to perform, risk-free procedure and is expected to become the major 3D imaging technique for oral tissue imprints in the near future. Several studies have confirmed that digital 3D dental models constructed through surface scanning technology accurately represent the original objects^2,3^. However, scanning of plaster dental models is also expected to have a major role in the construction of 3D oral tissue digital imprints, mainly for archiving existing physical dental models or when direct intraoral scanning is not possible. In such cases, conventional plaster dental models are constructed and then scanned with a surface scanner in order to create the digital model, similar to the one obtained intraorally.

Serial 3D dental models of an individual are regularly used in orthodontics, as well as in other medical and dental disciplines, to assess growth, treatment progress, or treatment outcome. This can traditionally be performed through the comparison of linear and angular measurements^4,5^. However, digital dental models offer great advantages with the capability to apply 3D superimposition techniques and to use the whole amount of 3D information for a thorough assessment and visualization of morphological changes^6^. For this purpose, two serial dental models of the same patient can be superimposed on a selected reference area that is considered anatomically stable. Then, changes in the neighboring structures can be precisely measured in three dimensions and visualized through color maps^6^.

So far, various techniques for 3D dental model superimposition have been proposed using the palate, tooth structures, the alveolar bone, or mini screws as superimposition references. The palatal structures, including the palatal rugae, are commonly used references due to their anatomical form stability and the additional advantage of being centrally located in the oral cavity^7–9^. In order to ensure proper registration of two serial dental models at the reference areas, the exact imprint of the region of interest is required. However, during the conventional impression and physical dental model construction process, surface artifacts, usually in the form of small (0.5–2 mm) bubble-type structures, can occur^10^. Such artifacts normally occur on morphologically complex areas, such as the palatal rugae, the gingival margins and the fissures of the teeth. Intraoral digital imprints also present artifacts, such as in areas with movable structures (e.g. non-attached mucosa) or in any other area, due to saliva or to the interference of undesirable structures during image acquisition of structures of interest. The inaccuracy of the digital model acquisition and creation process itself is also a factor that generates artifacts. Indeed, previous studies reported that both conventional and digital intraoral imprints show artifacts or inaccuracies^3,11,12^. Since such artifacts alter the original surface morphology of any structure of interest, this might have an influence on the superimposition outcomes.

Therefore, the aim of the present study was to assess the effect of artifacts on the superimposition outcome of serial 3D dental models, using three previously used palatal areas as superimposition references^1^. For this purpose, we assessed the superimposition performance on serial original plaster dental models of orthodontically treated adult patients and then repeated the whole process after cleaning the models by manual removal of visible artifacts. Furthermore, we scanned the cleaned models twice and repeated the superimpositions in order to assess a potential effect of the digital model generation process, which would be another source of artifacts.

## Methods

Ethical approval was obtained by the Institutional Ethics and Research Committee of the 251 Hellenic Airforce Hospital, Athens, Greece (approval number: 076/10571/16.06.2018). The methods were carried out in accordance with the relevant guidelines and regulations. All participants signed an informed consent prior to the use of their data in the study.

### Sample

The material for this study consisted of existing pre- (T0) and post-orthodontic treatment (T1) maxillary dental casts of 10 adult patients (2 males and 8 females; median age at T0: 27.4 years, range: 22.1–35.7 years) retrieved consecutively from the archive of a private orthodontic clinic in Cologne, Germany. The patients were treated between November 2013 and December 2017. The median time lapse between two serial dental models was 15.1 months (range: 9–27 months). This material was selected because these patients had no growth, no appliances in the palate and no extreme morphological changes due to treatment. For the needs of the study, 10 dental models that all presented visible artifacts were used. The detailed inclusion criteria are provided in Supplementary Table S1.

The dental models were obtained according to the regular protocol of the practice^1^ through alginate imprints, which were poured with plaster the same day. The plaster was mixed using an automated mixing device.

The dental casts were scanned with an intraoral 3D surface scanner (CS 3600, Carestream Dental) to obtain the 3D maxillary virtual models for the study, which consisted of approximately 115.000 vertices, each. All the original plaster dental models were scanned once using the same process that is used for intraoral scanning. After the first scanning, all the visible plaster artifacts on the palate and the teeth were manually removed by the first author according to the current practice by using appropriate instruments, such as wax knifes, in order to restore the original anatomy. These were bubble type artifacts, primarily of small size (0.5–2 mm), extending from the original plaster surface, which was also verified by comparing subsequent models of the same patient. Afterwards, the cleaned dental models where scanned once more with the same intraoral scanner, using the same process, in order to assess the effect of the scanning inaccuracy derived artifacts on the superimposition outcome.

### Superimposition procedure

Three different superimposition techniques were performed through Viewbox 4 software (version 4.1.0.1 BETA, dHAL Software, Kifissia, Greece), on three different palatal areas, as described below. Exactly the same protocol was applied for the original and the cleaned models.

#### Superimposition reference areas and the teeth of interest

Based on the results of a previous study^1^, the following three superimposition reference areas were selected on the T0 model: (A) a small area of the palate including the medial 2/3 of the third rugae and the area 5 mm dorsal to them, (B) area A, plus a 6 mm wide stripe on the midpalatal suture extending posteriorly to the level of a line connecting the lingual grooves of the 1^st^ permanent molars, and (C) almost the whole palate delimited by a line 5 mm distant from all gingival margins and extending posteriorly until the middle of the 1^st^ permanent molars (Supplementary Fig. 1). Superimpositions on areas A^7^ and B^13^ have been shown to work satisfactorily^1^, whereas varying results have been published regarding area C, in growing patients^1,14^. However, we also tested area C, since our study concerns non-growing patients.

Furthermore, in the T0 model, the clinical crown of the left central incisor and the right and left first permanent molars were selected as the teeth where movement from T0 to T1 was assessed^1^.

#### Implementation of the superimpositions

The present superimposition protocol consists of a slight modification of a previously tested and reported protocol^1,6^. The T0 and T1 3D models of each patient were superimposed on each reference area, using the software’s implementation of the iterative closest point algorithm (ICP)^15^ at two different settings. The basic setting (1) was: 100% estimated overlap of meshes, matching point to plane, exact nearest neighbor search, 100% point sampling, 50 iterations, and the alternative setting (2) was: the same as (1), but with 96% estimated overlap of meshes.

Following each superimposition of T0 and T1 models, the pre-selected teeth of interest at T0 were superimposed individually to the respective teeth at T1, as previously published^1,6^. This way, the positional changes of each tooth crown from T0 to T1 were recorded in three dimensions, with the origin of the axis positioned on each crown centroid^16^ and the axes of movement parallel to the midline palatal suture (Y: anteroposterior movement; positive: anterior) and on the occlusal plane (X: lateral movement; positive: right) or vertical to it (Z: vertical movement; positive: up/apical). Rotation of each tooth around the X (torque, positive: buccal crown), Y (tip, positive: left), and Z (rotation, positive: right buccal) axis was also recorded.

### Accuracy, precision, and reproducibility

After selecting the reference areas and teeth of interest, the first author repeated all superimpositions after a washout period of 2 weeks, to test intra-observer error. Further superimpositions were performed by the same researcher, one month later, on the 2^nd^ set of scans of the cleaned dental models, using setting 1, to assess the single effect of scanning inaccuracy on superimposition outcome, which was considered another source of error (artifacts). Identical reference areas were selected on the models of the same patient to avoid any effect of this factor on the results. For this purpose, the corresponding 3D models were superimposed and made transparent so that the operator could view the first selection and reproduce it. Accuracy and precision were assessed in a similar manner to a previous report^1^. To evaluate the accuracy obtained by each superimposition technique (areas A, B, and C), we assessed the congruence of the two models in area A by measuring the mean absolute distance (MAD)^17^ of each mesh vertex of the T0 model to the T1 surface. To attain a valid comparison between the techniques, only the vertices within area A, the anatomically most stable area^5,6,18–21^, were considered in the computation, irrespective of the area used for superimposition. However, the MAD was additionally computed for each reference area, to test differences between the two ICP parameter settings. Furthermore, MAD of areas A, B, and C when superimposing original dental models were compared to those from the superimposition of cleaned models, and associated colour maps were created.

The precision of each superimposition technique in measuring tooth movement was evaluated through the assessment of positional changes of three teeth of interest (the left central incisor and two first permanent molars). These variables are influenced by the accuracy of each superimposition technique and represent the main clinically relevant outcome. These outcomes were compared between the original and the cleaned models.

Reproducibility was tested through the assessment of intra-observer error on repeated measurements of variables used for accuracy and precision assessment.

### Statistical analysis

Statistical analysis was carried out by using the IBM SPSS statistics for Windows (Version 25.0. Armonk, NY: IBM Corp) and PERMANOVA^22,23^ software similarly to a previous relevant study^1^.

Raw data were tested for normality through the Kolmogorov-Smirnov and Shapiro-Wilk tests and did not have a normal distribution in certain cases. Thus, non-parametric statistics were applied.

Differences in the measured variables were evaluated using permutational multivariate analysis of covariance (MANCOVA), with factorial fixed or mixed effects models. Patient was set as a covariate in all cases to account for possible matching and clustering effects. Pair-wise a posteriori comparisons (permutation of residuals under a reduced model, after fitting covariables) were performed when significant differences were detected by the multivariate model.

Differences in accuracy between the two sets of settings were assessed by testing four crossed factors and their possible interactions: superimposition technique (fixed factor; 3 techniques), setting (fixed factor; 2 sets of settings), artifact status (fixed factor; 2 status), and time (fixed factor; 2 time points). The MAD between models in the most stable area (A) was the variable tested. Differences in the distance of each reference area with the two settings were pairwise tested for original and cleaned models, separately, using the Wilcoxon signed-rank test. For precision testing, five crossed factors and their possible interactions were analyzed: superimposition technique (fixed factor; 3 techniques), tooth (random factor; 3 teeth), setting (fixed factor; 2 sets of settings), artifact status (fixed factor; 2 status), and time (fixed factor; 2 time points). All vectors of positional change of each tooth were considered as dependent variables (6 vectors: x-lateral movement, y- anteroposterior movement, z-vertical movement, x-torque, y-tip, z-rotation).

Permutational MANCOVA was done on Euclidean distances calculated from raw data. The P-value was calculated on raw data through permutation of residuals under a reduced model, with 9999 random permutations for accuracy testing and 999 random permutations for precision testing. In cases when there were few unique permutations possible, Monte Carlo asymptotic p-value was used instead^22^.

In all cases, a two-sided significance test was carried out at an alpha level of 0.05. Bonferroni correction was applied for pairwise a posteriori multiple comparison tests.

To overcome potential limitations of methods described above^24^ the Bland-Altman method (difference plot)^25^ was also used to evaluate agreement between original and cleaned models in the assessment of tooth movement for each single case. Differences between repeated original model superimposition results with all superimposition techniques were also assessed through the Bland-Altman method (difference plot), as well as differences between superimposition outcomes of the first versus the second scan of the cleaned models.

## Results

The main effects of different settings, cleaning status (artifact removal) and time points on accuracy measurements were not significant, as well as any interaction of these factors (Table 1). The superimposition technique was the only factor that affected accuracy measurements, with techniques A and B showing varying accuracy compared to technique C (Table 1). As already shown in a previous study, techniques A and B showed higher accuracy than technique C (Table 2)^1^.

**Table 1:** Non parametric MANCOVA on accuracy measurements (deviation between structures) performed with different settings, cleaning status, and measurement time points.

Factor	d.f.	F	P
Covariate (patient)	1	31.293	0.000*
Superimposition	2	18.269	0.000*
Setting	1	0.712	0.396
Cleaning	1	0.001	0.981
Time	1	0.017	0.897
Superimposition x Setting	2	0.347	0.703
Superimpostion x Cleaning	2	0.035	0.967
Superimposition x Time	2	0.005	0.994
Setting x Cleaning	1	0.014	0.908
Setting x Time	1	0.000	0.993
Cleaning x Time	1	0.001	0.971
Superimposition x Setting x Cleaning	2	0.069	0.934
Superimposition x Setting x Time	2	0.027	0.973
Superimposition x Cleaning x Time	2	0.019	0.981
Setting x Cleaning x Time	1	0.021	0.885
Superimposition x Setting x Cleaning x Time	2	0.005	0.995
Residual	215		
Total	239		
**Comparison** ^a^	**t**		**p – value**
A vs. B	1.232		0.218
A vs. C	5.913		0.000*
B vs. C	5.128		0.000*

Four crossed factors and their interactions were analyzed in each case having “patient” as a covariate: superimposition technique (fixed factor; 3 techniques), setting (fixed factor; 2 settings), cleaning (fixed factor; 2 status), and time (fixed factor; 2 measurement time points).

9999 permutations.

*p < 0.05.

^a^*a posteriori* pairwise tests between superimposition techniques.

A, B, C correspond to the three superimposition techniques and reference areas tested in the study.

**Table 2:** Accuracy values of each superimposition technique in the different cases studied.

	A	B	C
Original – Setting 1 – M1	0.060 (0.03)	0.072 (0.04)	0.098 (0.04)
Original – Setting 1 – M2	0.060 (0.03)	0.072 (0.04)	0.098 (0.04)
Cleaned – Setting 1 – M1	0.061 (0.06)	0.070 (0.05)	0.102 (0.06)
Cleaned – Setting 1 – M2	0.061 (0.06)	0.070 (0.05)	0.096 (0.04)
Original – Setting 2 – M1	0.063 (0.05)	0.071 (0.03)	0.090 (0.02)
Original – Setting 2 – M2	0.063 (0.03)	0.071 (0.03)	0.094 (0.02)
Cleaned – Setting 2 – M1	0.061 (0.05)	0.070 (0.04)	0.098 (0.05)
Cleaned – Setting 2 – M2	0.060 (0.05)	0.070 (0.04)	0.098 (0.05)

Values represent median (interquartile range) of mean absolute distance (MAD) between corresponding form-stable structures (area A) in millimeters (n = 10 patients).

A, B, C correspond to the three superimposition techniques and reference areas tested in the study.

M: Measurement time point.

When the MAD values between corresponding reference areas were tested, no specific effect was evident, except from a negligible difference between original and cleaned dental models in area C (Table 3).

**Table 3:** Congruence of reference areas in the different cases studied.

	A	B	C
Setting 1 – Original – M1	0.060 (0.03)	0.071 (0.04)	0.117 (0.08)
Setting 2 – Original – M1	0.063 (0.05)	0.068 (0.04)	0.117 (0.08)
Setting 1 – Original – M2	0.060 (0.03)	0.071 (0.04)	0.117 (0.08)
Setting 2 – Original – M2	0.063 (0.03)	0.069 (0.04)	0.117 (0.08)
Setting 1 – Cleaned – M1	0.061 (0.06)	0.066 (0.05)	0.124 (0.10)
Setting 2 – Cleaned – M1	0.061 (0.05)	0.067 (0.05)	0.124 (0.10)
Setting 1 – Cleaned – M2	0.061 (0.06)	0.066 (0.05)	0.124 (0.10)
Setting 2 – Cleaned – M2	0.060 (0.05)	0.067 (0.05)	0.124 (0.10)
p-value*	0.524^a^	0.664^a^	0.021*^a^

Values represent median (interquartile range) of mean absolute distance (MAD) between corresponding reference areas (A, B, or C) used each time in millimeters (n = 10 patients).

^a^Friedman test.

*p < 0.05.

A, B, C correspond to the five superimposition techniques and reference areas tested in the study.

M: Measurement time point.

Regarding precision of tooth movement measurements that occurred from pre- to post-treatment, the main effects of different teeth, settings, cleaning status (artifact removal), and time points were not significant, as well as their interactions, except for four cases that were considered negligible (Table 4). The superimposition technique had a significant effect on tooth movement measurements, with all techniques differing from each other significantly (Table 4).

**Table 4:** Non parametric MANCOVA on precision measurements (tooth movement) performed with different settings, cleaning status, and time points.

Operator factor	d.f.	F	P
Covariate (patient)	1	14.609	0.001*
Superimposition	2	13.281	0.016*
Tooth	2	1.105	0.328
Setting	1	0.032	0.993^a^
Cleaning	1	0.045	0.993^a^
Time	1	0.007	1.000^a^
Superimposition x Tooth	4	0.145	1.000
Superimposition x Setting	2	0.366	0.823
Superimposition x Cleaning	2	1.725	0.208
Superimposition x Time	2	0.438	0.814
Tooth x Setting	2	1.968	0.095
Tooth x Cleaning	2	1.462	0.169
Tooth x Time	2	1.579	0.152
Setting x Cleaning	1	0.001	0.892
Setting x Time	1	0.006	0.883
Cleaning x Time	1	0.024	0.910
Superimposition x Tooth x Setting	4	0.211	0.993
Superimposition x Tooth x Cleaning	4	0.031	1.000
Superimposition x Tooth x Time	4	0.023	1.000
Superimposition x Setting x Cleaning	2	0.361	0.872
Superimposition x Setting x Time	2	0.457	0.835
Superimposition x Cleaning x Time	2	1.389	0.322
Tooth x Setting x Cleaning	2	4.788	0.003*
Tooth x Setting x Time	2	4.833	0.001*
Tooth x Cleaning x Time	2	4.279	0.004*
Setting x Cleaning x Time	1	0.009	0.951
Superimposition x Tooth x Setting x Cleaning	4	0.069	1.000
Superimposition x Tooth x Setting x Time	4	0.040	1.000
Superimposition x Tooth x Cleaning x Time	4	0.076	1.000
Superimposition x Setting x Cleaning x Time	2	0.806	0.543
Tooth x Setting x Cleaning x Time	2	13.700	0.001*
Superimposition x Tooth x Setting x Cleaning x Time	4	0.110	1.000
Residual	647		
Total	719		
**Comparison** ^b^		**t**	**p-value**
A vs. B		4.618	0.002*^a^
A vs. C		2.922	0.022*^a^
B vs. C		3.358	0.019*^a^
**Comparison** ^c^		**t**	**p-value**
Setting 1, Incisor: Cleaned vs. Original		0.664	0.650
Setting 1, Molar R: Cleaned vs. Original		2.136	0.009**
Setting 1, Molar L: Cleaned vs. Original		1.319	0.164
Setting 2, Incisor: Cleaned vs. Original		1.434	0.127
Setting 2, Molar R: Cleaned vs. Original		0.777	0.556
Setting 2, Molar L: Cleaned vs. Original		2.201	0.012
**Comparison** ^d^		**t**	**p-value**
Time 1, Incisor: Cleaned vs. Original		0.586	0.733
Time 1, Molar R: Cleaned vs. Original		1.829	0.032
Time 1, Molar L: Cleaned vs. Original		1.3576	0.159
Time 2, Incisor: Cleaned vs. Original		1.432	0.125
Time 2, Molar R: Cleaned vs. Original		0.587	0.733
Time 2, Molar L: Cleaned vs. Original		2.229	0.008**

Five crossed factors and their possible interactions were analyzed in each case having patient as a covariate: superimposition technique (fixed factor; 3 techniques), tooth (random factor; 3 teeth), setting (fixed factor; 2 settings), cleaning (fixed factor; 2 status), and time (fixed factor; 2 measurement time points). All vectors of positional change of each tooth were considered as dependent variables (6 vectors: x-lateral movement, y-anteroposterior movement, z-vertical movement, x-torque, y-tip, z-rotation).

999 permutations.

*p < 0.05.

^a^Monte Carlo asymptotic p-value.

^b^Tests among levels of the factor Superimposition.

^c^Tests among levels of the factor Cleaning within levels of factors Setting and Tooth.

^d^Tests among levels of the factor Cleaning within levels of factors Time and Tooth.

A, B, C correspond to the five superimposition techniques and reference areas tested in the study.

**Figure 1:**
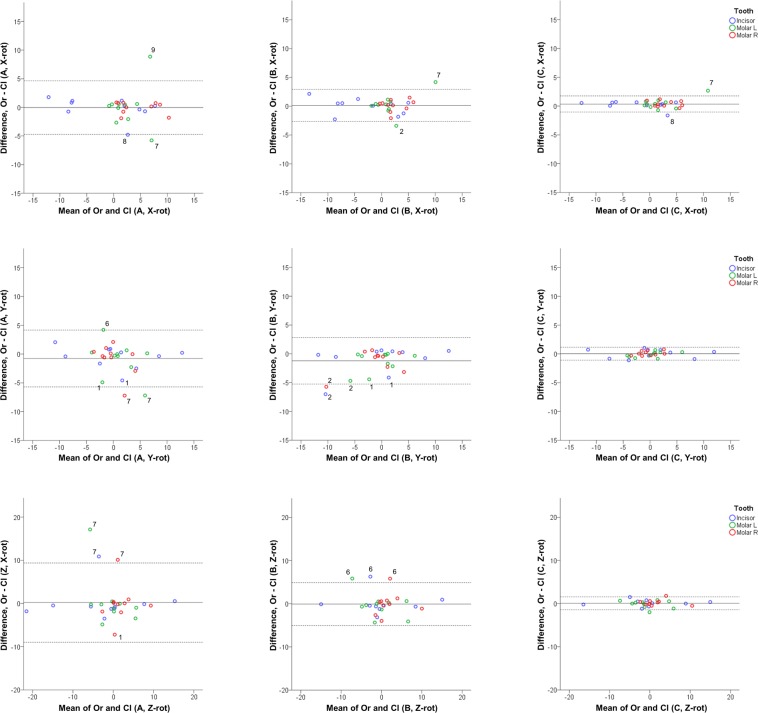
Differences between original and cleaned dental model superimposition results with techniques (**A**–**C**). Bland Altman plots of differences of (**A**–**C**) superimposition techniques in original and cleaned models superimposed with setting 1, at Μ1 (measurement time point 1). These refer to the measured tooth rotations of the three teeth of interest in the three planes of space (°). The axes length represents the true range of observed values of structural changes. The continuous horizontal line shows the mean and the dashed lines the 95% confidence intervals. Point labels represent patients with values located outside the 95% confidence intervals of each set of measurements.

**Figure 2:**
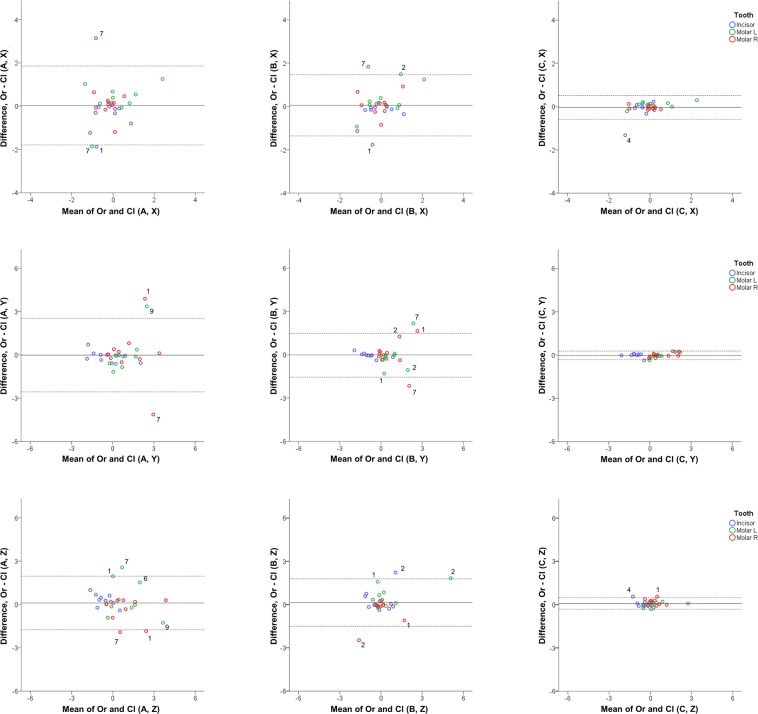
Differences between original and cleaned dental model superimposition results with techniques A, B, and C. Bland Altman plots of differences of A, B, and C superimposition techniques in original and cleaned models superimposed with setting 1, at Μ1 (measurement time point 1). These refer to the measured tooth movements of the three teeth of interest in the three planes of space (mm). The axes length represents the true range of observed values of structural changes. The continuous horizontal line shows the mean and the dashed lines the 95% confidence intervals. Point labels represent patients with values located outside the 95% confidence intervals of each set of measurements.

**Figure 3:**
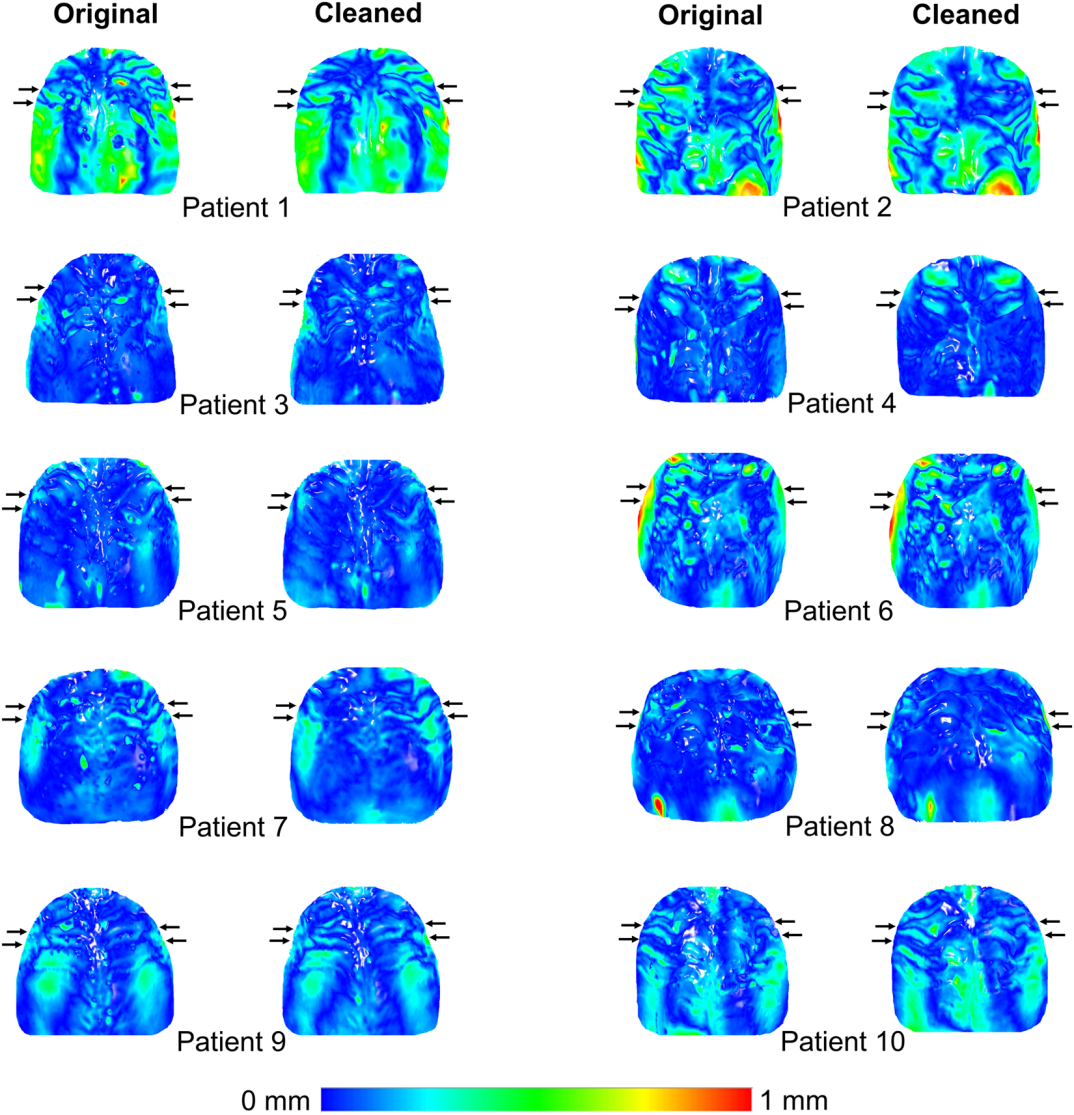
Color maps showing morphological differences in the palate between superimposed pre- and post-treatment original and cleaned models in area C. Superimposition of pre- (T0) and post-treatment (T1) models of each patient, before and after cleaning, in area C, with setting 1, at M1 (measurement time point 1). The extent of congruence between serial models in each case is shown with specific color coding. In each case, the upper pair of black arrows shows the position of the second rugae and the lower pair the third rugae. Note that despite the presence of artifacts that can be identified as localized differences in the colour maps, the overall pattern remained unaltered. This can explain why superimposition outcomes of area C were not considerably affected by the artifact removal process. Note also that patients 1 and 2 showed considerably larger changes in their palates from T0 to T1, compared to the other patients, but the overall colour pattern did not change considerably due to artifact cleaning also in this case. These differences can be due to treatment effects or to model creation inaccuracies, such as in the large red mark in the posterior palate of patient 2.

**Figure 4:**
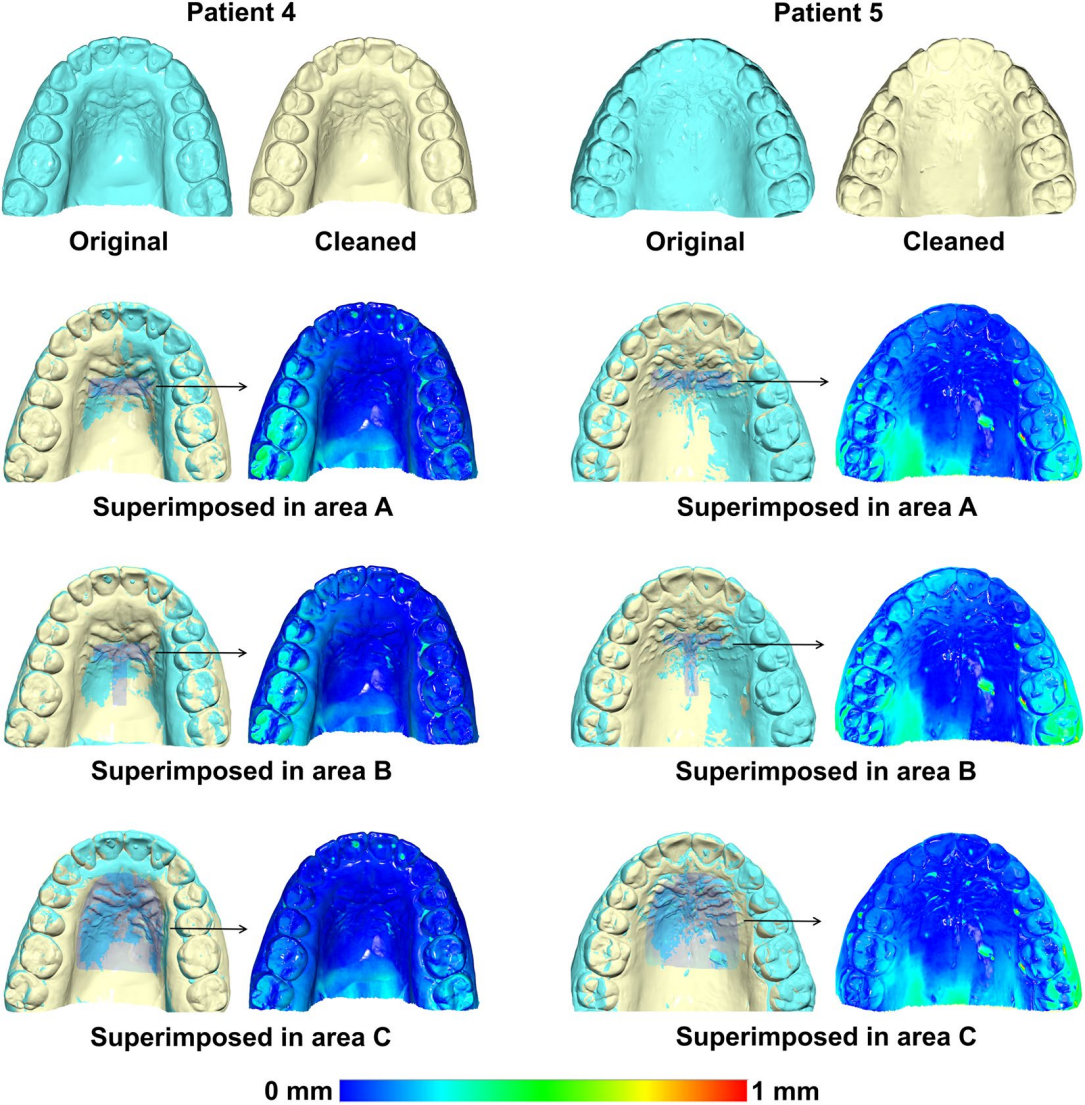
Examples of original and cleaned post-treatment (T1) models superimposed on the three reference areas used in the study. As shown in the respective colour maps, Patient 4 represents a case with few artifacts in the superimposition reference areas, where the artifact removal already exerted an effect on the superimposition of the original with the subsequent cleaned model. Patient 5 represents a case with more artifacts, and these exerted a greater effect on the superimposition. This differential effect, depending on the amount of artifacts, primarily existed for areas A and B, but not for C, which was the largest one. Superimpositions were performed with setting 1, at M1 (measurement time point 1).

One-sample t-tests showed that mean differences between original and cleaned models with each superimposition technique (A, B, and C) in detected tooth movements (precision) were not significantly different from 0 in any case (p < 0.002; Bonferroni correction applied) (Supplementary Table S2). However, although the mean effects of the above investigated factors were not significant, Bland Altman plots revealed individual differences between measurements that showed a significant effect of artifact removal on the detected tooth movements, in all the components tested (Figs 1 and 2). With both settings, the smaller the selected reference area, the higher the effect of artifacts in the detected tooth movements. Pair-wise *a posteriori* tests (*Wilcoxon signed-rank test)* between measurements in original and cleaned models showed that techniques A and B did not differ significantly from each other, but both of them were different from technique C (p < 0.01; Bonferroni correction applied). For area C, which was the largest area tested in the study, the effect of artifact removal on measured tooth movement was not considered clinically significant (Figs 1 and 2; Supplementary Tables S2 and S3). When superimposing pre- and post-treatment original and cleaned models in area C, it was evident that despite the presence of artifacts that can be identified as localized differences in the colour maps, the overall colour pattern of the palate remained unaltered (Fig. 3). Figure 4 shows two examples where the superimposition outcomes of area C were not considerably affected by the artifact removal process. There was no evidence that the extent of difference between original and cleaned models in the measured tooth movements was related to the extent or the direction of tooth movement in either case (Figs 1 and 2).

In order to assess if the effect of artifact removal on the detected tooth movement was due to this factor solely, further Bland Altman plots were performed, to test the effect of the software and the operator on repeated individual measurements of the same sets of models. They revealed perfect repeatability of the process, apart from very few cases considering area A (Supplementary Fig. 2).

In the same manner, we tested if the model acquisition process itself had an effect on the superimposition outcome, by performing a second scan of the cleaned models. Thereafter, the detected tooth movements through superimposition of the first and the second sets of cleaned model scans were compared and Bland Altman plots revealed considerable differences (Supplementary Fig. 3). The magnitude of the effect of the dental model acquisition process on the detected tooth movement was less than the effect of cleaning (Supplementary Tables S3 and S4), but it was not considered negligible. This is also illustrated when comparing Fig. 4 to Supplementary Fig. 4, showing two cases with variable amounts of artifacts.

## Discussion

The present study is the first to thoroughly assess the effect of surface artifacts on the superimposition outcome of serial 3D dental models. Indeed, significant differences in individual tooth movement measurements were evident due to the presence of artifacts. Under clinical conditions, such artifacts are to be expected, both with conventional and digital impression techniques^3,10–12^. The present study showed that even the imprecision of the digital model generation process itself can have a considerable effect on the detected tooth movement measurements. Our findings highlight the importance of having accurate, artifact-free models, in order to obtain a valid and precise assessment of the tooth movement that occurred between two time points, through serial 3D patient model superimposition.

In accordance to previously published results^1^, the effect of all the factors tested in the present study on the detected tooth movement tended to decrease with an increase of the size of the superimposition reference area. However, previous studies have shown that only a small palatal area remains relatively stable during growth and treatment^7,8,18–21^. So, if we consider that small stable reference areas are required for serial dental model superimpositions to obtain a valid assessment of changes that occur during treatment and growth, the need for accurate and artifact-free dental models becomes essential.With the increasing incorporation of 3D-imaging software and hardware tools in everyday clinical practice, it is expected that the interest for proper superimposition techniques will considerably expand in the next few years. However, to facilitate the development and proper use of these techniques, deep understanding of the way they work and the factors that may affect their outcomes is indispensable. So far, several studies have tested the accuracy of intraoral scanners both *in vivo* and *in vitro* and concluded that relatively precise 3D dental model representations of a patient’s mouth can be performed^3,26–29^. However, the present study showed that even a small imprecision of the intraoral scanner can significantly affect tooth movement assessment. It should be considered that by performing a superimposition of two dental models, the surface morphology of both models affects the outcome. Thus, even if both models, as single items, present only few inaccuracies or artifacts, when superimposed to each other, the combined effect might be larger than expected. Since plaster artifacts are expected to be different in amount, form, and distribution in two serial dental models, and thus, differently alter the reference areas, superimposition of cleaned models might be considered to provide results of higher trueness.

The present study design simulated actual clinical conditions, where different types of artifacts exist. For this reason, we compared the superimposition outcome of original plaster dental models with that of cleaned models, after performing the common cleaning process that the dental technicians do before delivering the dental models. Such artifacts are commonly present on the plaster dental models due to imperfections of the imprint procedure, to entrapment of air into the plaster material during the mixing process, or due to water clinging to the pattern. Any of these occurrences can affect the original anatomy of the represented structures^30,31^ and therefore, it is a common practice to perform the cleaning process before using the models. Through this simulation, we studied a certain type of artifact, but the results are expected to generalize to other types of artifacts of similar extent, such as those expected from digital model creation^3^.

Apart from the artifacts of the physical plaster dental models, the inaccuracy of the intraoral scanner itself could be an artifact causing factor. In the initial artifact removal evaluation, two scans of the same model - before and after cleaning - were performed. Thus, the scanner imprecision effect was integrated in the artifact removal effect. To assess the isolated effect of the scanner imprecision factor on the results, we tested the differences in outcomes between pre- and post-treatment superimpositions of repeated cleaned dental model scans. Although, it has been previously shown, that the reference area selection by different operators had no significant effect on the superimposition outcome^1^, identical reference areas and teeth of interest to those of the first evaluation have been selected, so that the only influential factor would be the digital model generation process. Indeed, the effect of the scanner inaccuracy on superimposition outcome was smaller than the one of the cleaning process, but anyhow it was considered clinically significant. The scanner used in the present study (CS 3600) has equivalent accuracy to the highest standards that the currently available intraoral scanners can reach^26,27^.

Other factors that could have influenced the results of the present study might be the software function, the different settings used, or the operator. Although a previous study did not show any significant effect of these factors on the results^1^, we performed similar tests to verify this. Indeed, the different settings did not affect the accuracy or the precision of tooth movement measurements, and the repeated assessments generated similar results at the first and second time point. Thereby, as already evident in previous studies^1,6^, the proper functioning of the software was confirmed.

Although the mean effect of artifacts on superimposition outcomes was negligible, Bland Altman plots revealed significant effects in individual cases. These effects were higher in certain cases than in others. Possible reasons might be the limited surface irregularities of the selected reference areas or the different amounts of artifacts. Other possible influential factors could be the shape, size, or location of the artifacts. This issue was investigated in an exploratory manner and thus requires further investigation. For example, when looking at individual patients in more detail, patient 7 consistently showed higher differences in the various measurements tested. This patient presented a smooth third rugae area, with few, shallow surface irregularities (Supplementary Fig. 5). Thus, it might be suspected that the proper registration of the corresponding dental models was difficult to be achieved by the best-fit algorithm, especially when superimposing on the smallest superimposition reference area. Patients 1 and 2 also presented higher differences, that could be attributed to the fact that these patients showed bigger changes in their palates from T0 to T1, as shown in Fig. 3.

## Limitations

No 3D models that would be identical to the actual anatomy of the patients were available, in order to compare these with the results from the used original and cleaned dental models. However, until now, such models are not possible to be obtained intraorally. Furthermore, the aim of the present study was to assess the effect of artifacts in the superimposition of regular dental models, and therefore, either the original or the cleaned dental models can be considered as the comparison group, fulfilling the needs of the study.

In the same line, the artifact removal process could also introduce other artifacts into the model, since no true 3D model was available, and thus, it was performed manually, according to the standard practice. To address this issue, apart from clinical experience of the operator, subsequent models of the same patient were compared to assess the original anatomy.

The present study included adult patients with no active growth, no extreme malocclusions and no extensive orthodontic treatment. Since these conditions might have influenced the tested effects, the usage of a more variable sample could have made the interpretation of the results more difficult. However, this encompasses the limitation, that the results might be modified in other patient groups with active growth, extreme malocclusions or extensive treatments and therefore, generalizability might be questioned.

The type of artifacts (e.g. shape, size, location) could be another factor that might affect generalizability. We simulated actual clinical conditions by removing physical artifacts from plaster models, which is a common practice in dentistry. We expect that our findings can be extrapolated to other type of artifacts, if they are of a similar extent, such as those present in digital intraoral scans, but this remains to be tested.

The sample size of 10 adult patients could be considered limited. However, in the present study, we decided to include only a small, but homogenous patient group and therewith, avoid various confounding factors that could have skewed the findings. The number of 10 patients was adequate for the purposes of the present study and provided statistically significant results for the main outcomes tested, implying also adequate power.

The software functioning and the intraoral scanner used for the study could comprise additional factors that might affect the findings. The software has already been validated through previous studies^1,6^ and it was further validated in this study and proved to perform adequately. The same applies to the intraoral scanner, which is commonly used in everyday practice and has been shown to work properly^26,27^. Therefore, we do not expect that any of these factors could limit the validity of our findings.

## Conclusion

The present study highlights the importance of accurate, artifact-free models, to obtain a valid and precise assessment of tooth movement through serial 3D model superimposition. The presence of regular artifacts on dental models substantially affected individual tooth movement assessment through superimposition of serial patient models. Furthermore, even the imprecision of a digital model generation itself can have a considerable effect in the detected tooth movements. Noteworthy, the effects tested in the present study tended to decrease with an increase of the reference area used for superimposition.

To generalize the findings of the study, further research should be performed in different patient groups, of different growth status and malocclusion severity. Additionally, the influence of different types of artifacts (e.g. in shape, size, and location) or of artifacts generated through direct intraoral scanning might be further investigated.

## Supplementary information

The effect of regular dental cast artifacts on the 3D superimposition of serial digital maxillary dental models

## Data Availability

The datasets generated and/or analyzed during the current study are available from the corresponding author on reasonable request.
