# Supplementary material for: The effect of regular dental cast artifacts on the 3D superimposition of serial digital maxillary dental models
Source: Sci Rep. 2019 Jul 19;9:10501. doi: 10.1038/s41598-019-46887-1 (PMC6642138; doi:10.1038/s41598-019-46887-1)
Supplement: Supplementary file 1 — The effect of regular dental cast artifacts on the 3D superimposition of serial digital maxillary dental models [file 41598_2019_46887_MOESM1_ESM.docx]

**The effect of regular dental cast artifacts on the 3D superimposition of serial digital maxillary dental models**

Eva Henninger, Georgios Vasilakos, Demetrios Halazonetis, Nikolaos Gkantidis

**Supplementary Figure 1.** Superimposition reference areas used in the study. **a.** The area of the palate limited anteriorly by the medial 2/3 of the third rugae and laterally by two lines parallel to the midpalatal suture and extending posteriorly 5 mm from the third rugae. **b.** Area A, plus a 6 mm wide stripe on the midpalatal suture extending posteriorly to the level of a line connecting the lingual grooves of the 1^st^ permanent molars at the gingival level. **c.** Almost the whole palate delimited by a line 5 mm distant from all gingival margins and extending posteriorly until a line connecting the lingual grooves of the 1^st^ permanent molars at the gingival level.


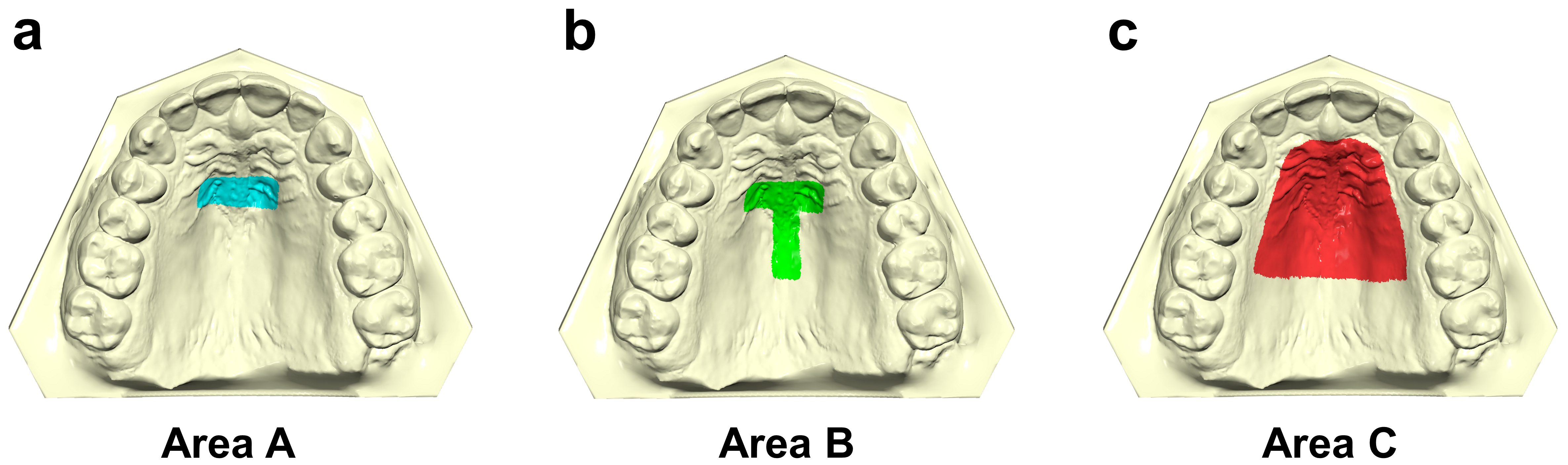

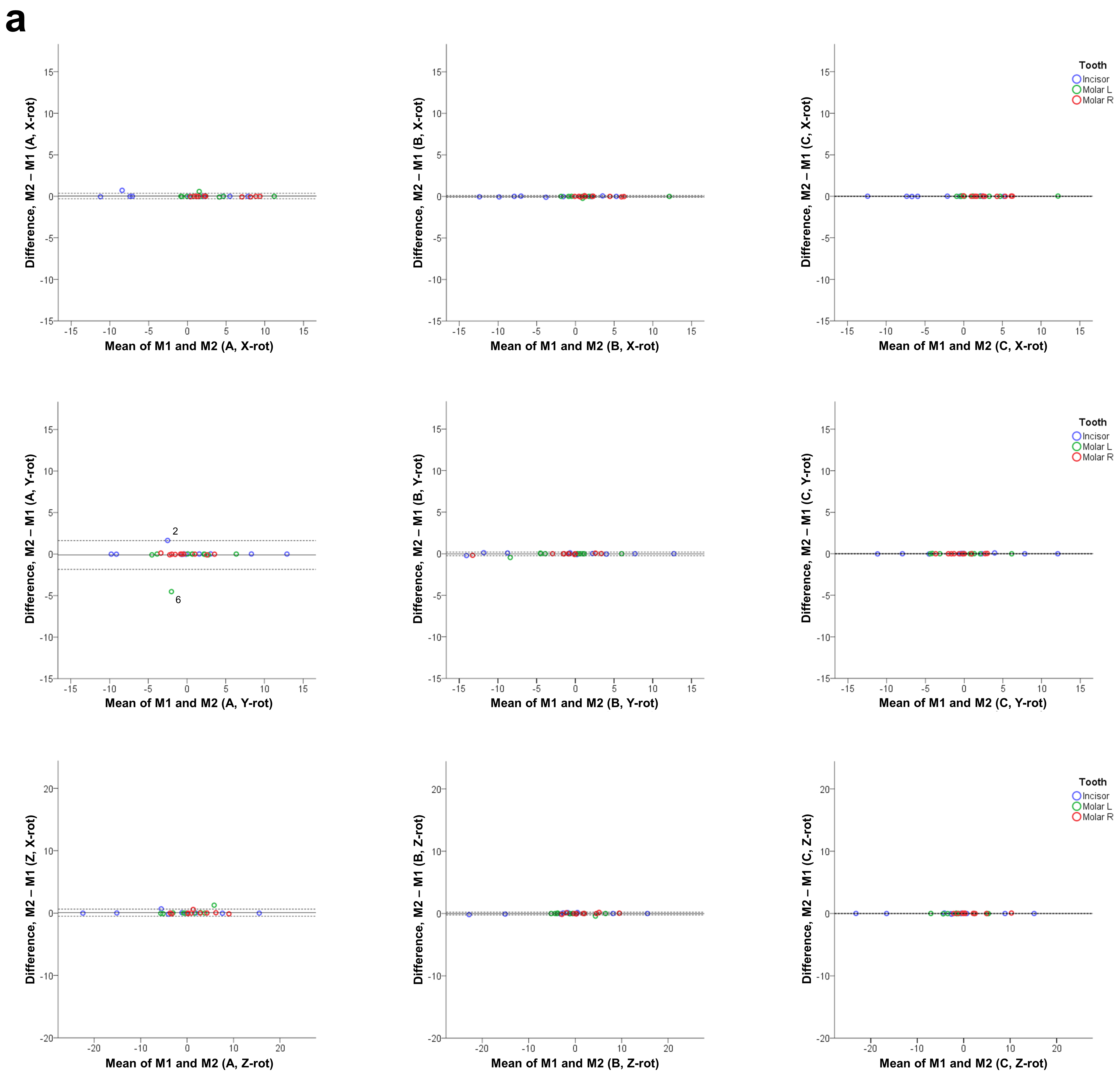


**Supplementary** **Figure 2.** Differences between M1 and M2 original model superimposition results with techniques A, B, and C. Bland Altman plots of differences of A, B, and C superimposition techniques performed at two time points (M1: measurement time point 1; M2: measurement time point 2), with setting 1. These refer to **a.** the measured rotations (°) and **b.** the movements (mm) of the three teeth of interest in the three planes of space. The axes length represents the true range of observed values of structural changes. The continuous horizontal line shows the mean and the dashed lines the 95% confidence intervals. Point labels represent patients with values located outside the 95% confidence intervals of each set of measurements.


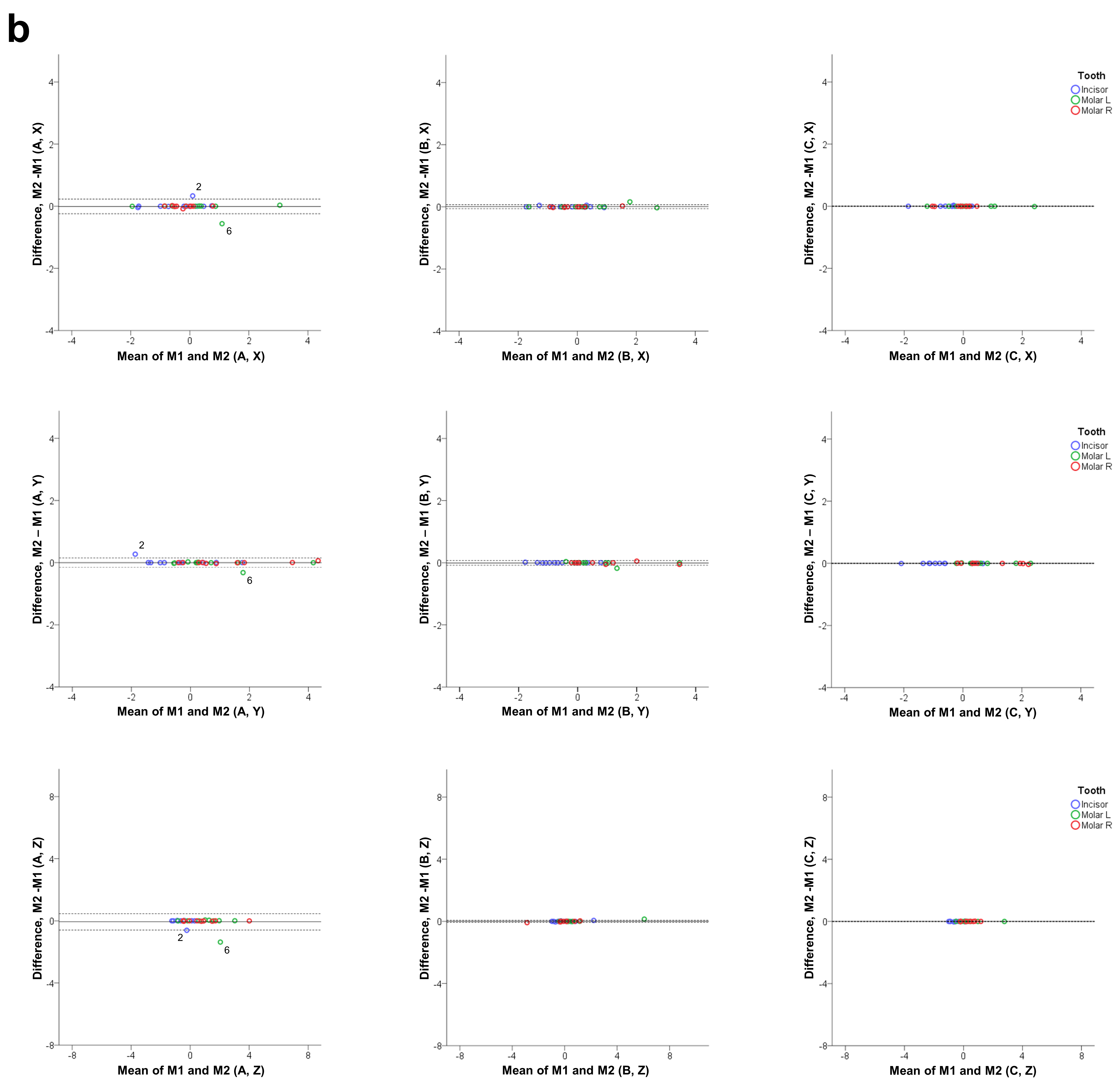


**Supplementary Figure 3.** Differences between Scan 1 and Scan 2 cleaned model superimposition results with techniques A, B, and C. Bland Altman plots of differences of A, B, and C superimposition techniques performed on two different set of scans of the same cleaned models (Scan 1: 1^st^ set of cleaned models; Scan 2: 2^nd^ set of cleaned models), with setting 1. These refer to **a.** the measured rotations (°) and **b.** the movements (mm) of the three teeth of interest in the three planes of space. The axes length represents the true range of observed values of structural changes. The continuous horizontal line shows the mean and the dashed lines the 95% confidence intervals. Point labels represent patients with values located outside the 95% confidence intervals of each set of measurements.


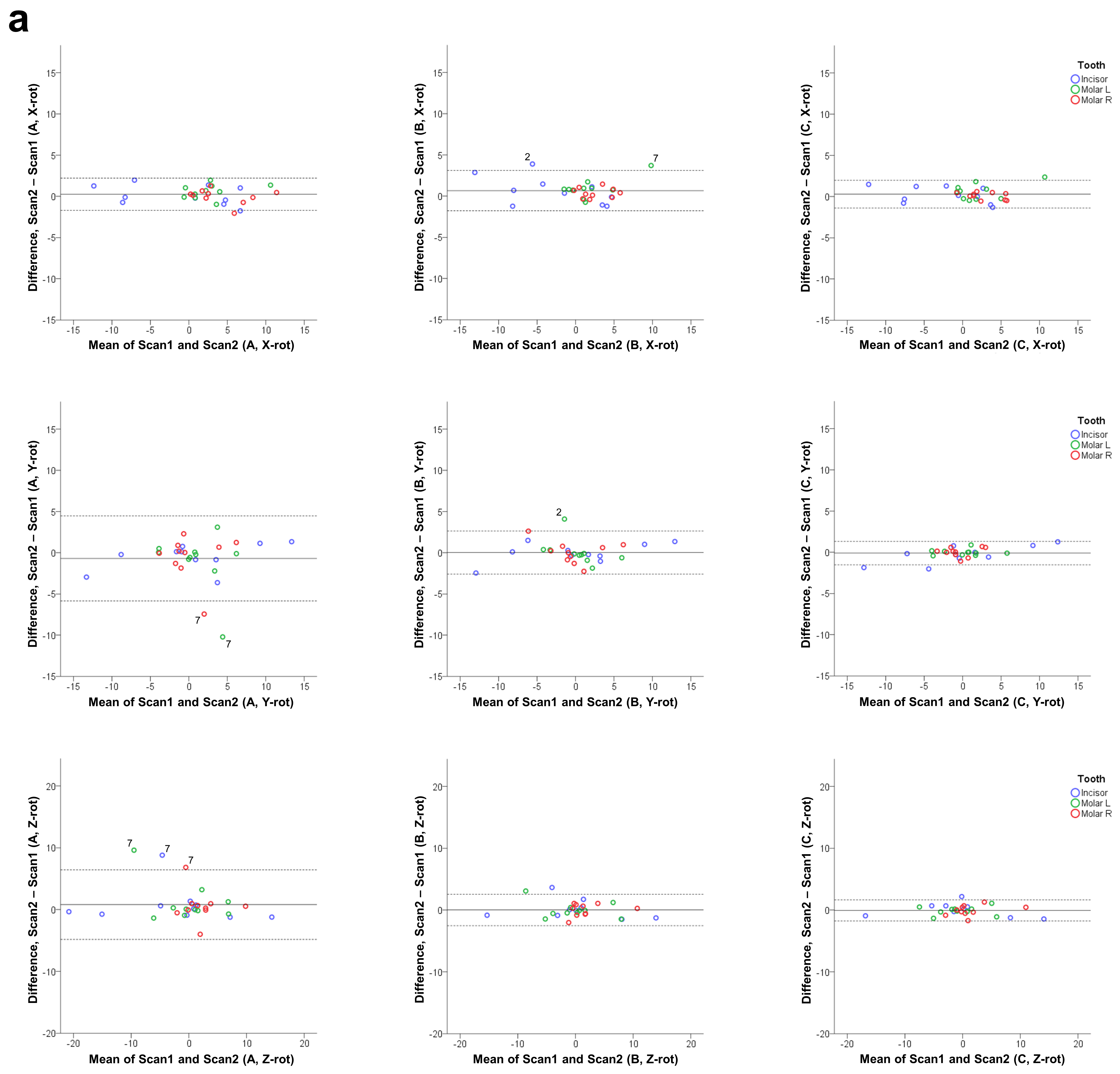

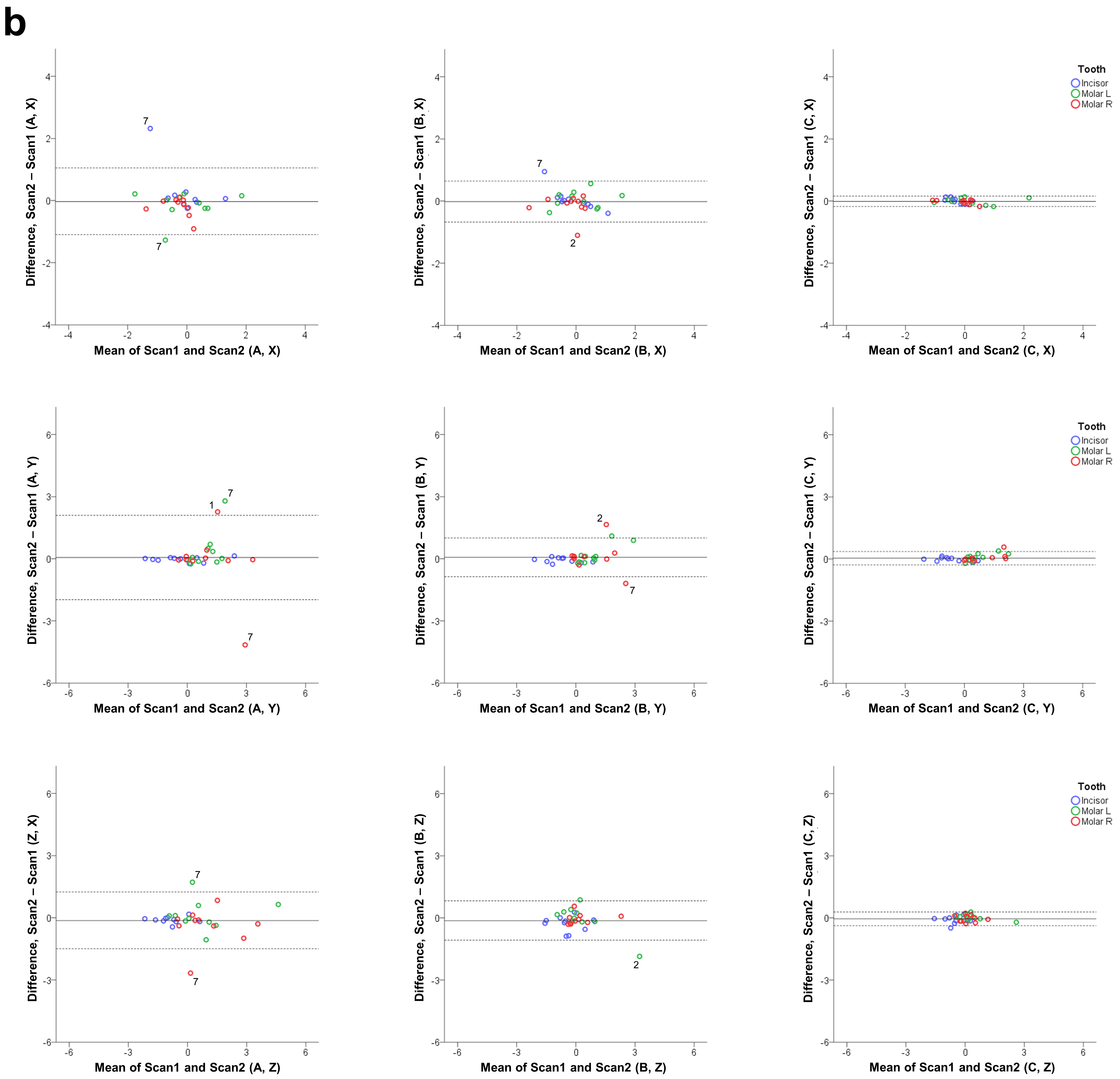


**Supplementary Figure 4.** Examples of repeatedly scanned cleaned post-treatment (T1) models superimposed on the three reference areas used in the study. The colour maps show that there is an effect of the model acquisition process itself, which is smaller than that shown in Figure 4. This implies that part of the effect shown in Figure 4 is attributed to the presence of artifacts and part to the image acquisition process itself. The effect of the image acquisition process was greater in patient 5 than in patient 4, indicating that this may have contributed on the differential effect shown in Figure 4. Once more the effect was reduced when superimposing in area C. Superimpositions were performed with setting 1.


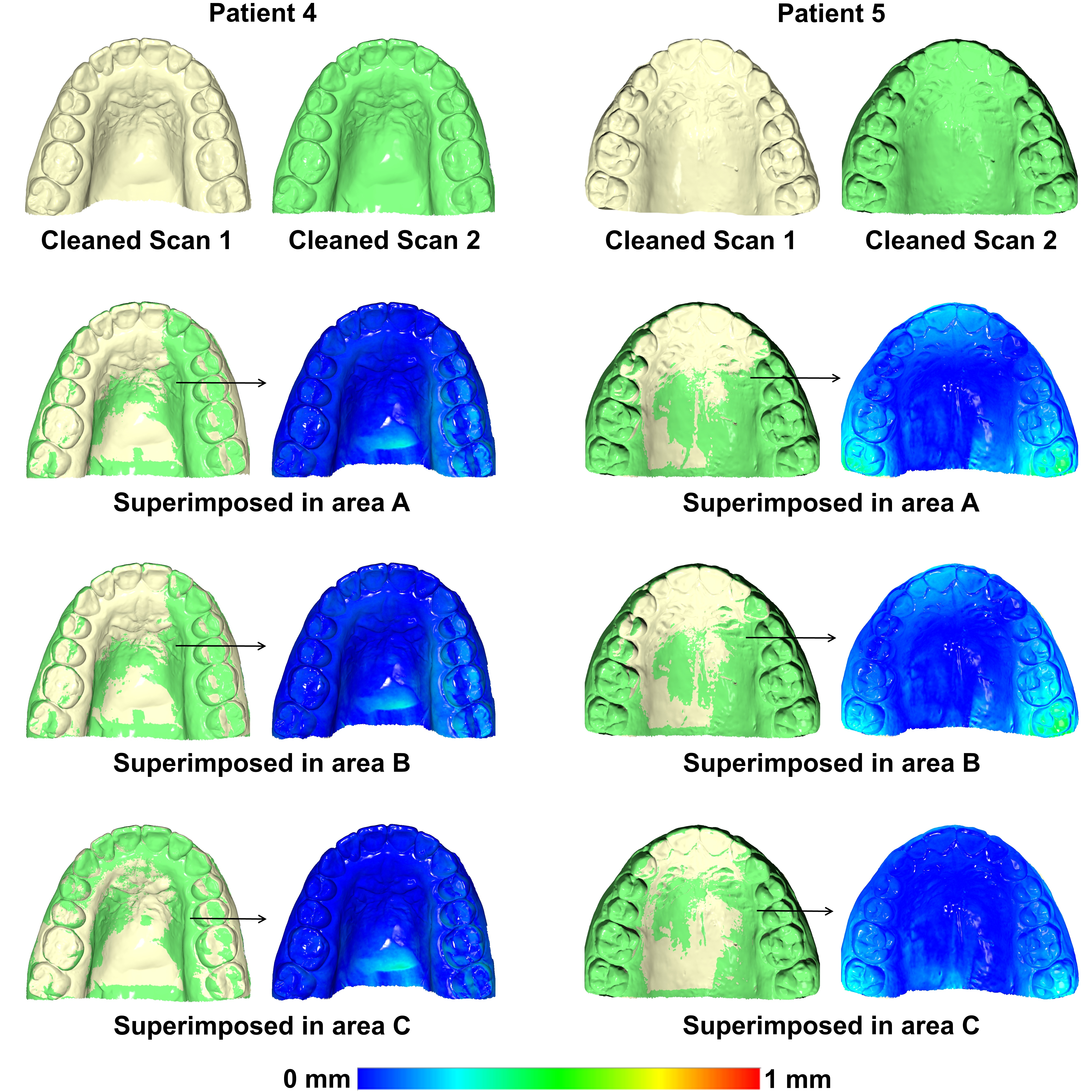

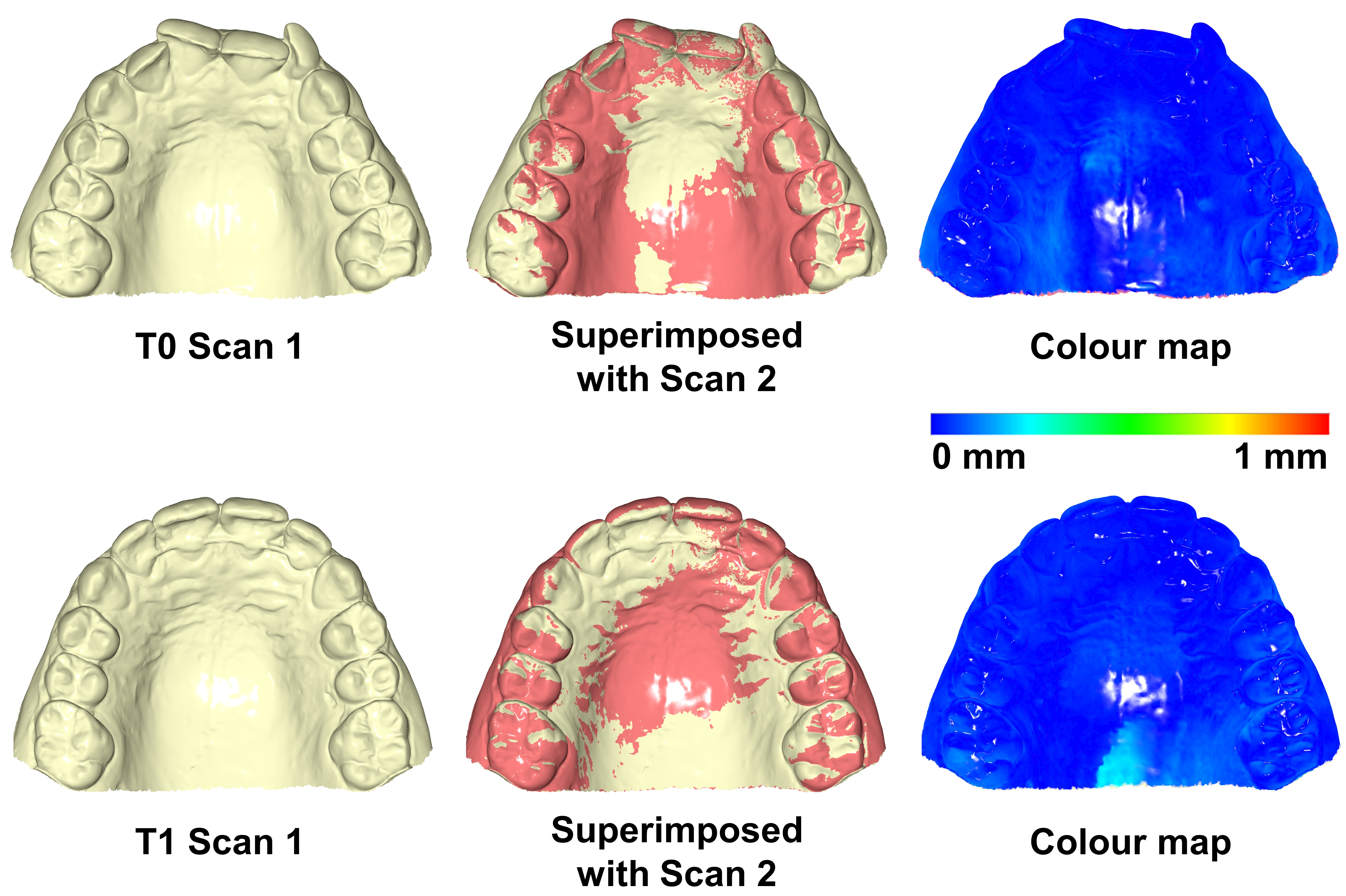


**Supplementary Figure 5.** Superimposition of two serial scans of cleaned dental models of patient 7. As shown in the respective colour maps, when the single models obtained from the first and second scan were superimposed on the whole surface, they showed negligible differences. Thus, we suspect that the reason of imprecise registration of the corresponding reference areas in this case, especially in areas A and B, is that this patient presents a smooth third rugae area, with few, shallow surface irregularities, which is difficult to be properly registered through the best-fit algorithm.

**Supplementary Table S1.** Inclusion criteria of the patients assessed in the present study.

| **Nr.** | **Criterion** |
| --- | --- |
| **1.** | Non-extraction orthodontic treatment with buccal fixed appliances in both jaws |
| **2.** | Minimum 1 year after the end of any previous orthodontic at treatment start |
| **3.** | Maximum 6 months from initial records to treatment start |
| **4.** | No appliance or intervention in contact with the palate (e.g. activator, mini-screws, Nance) at least 1 year prior to and during treatment |
| **5.** | No missing teeth from 2^nd^ molar to 2^nd^ molar |
| **6.** | Treatment duration between 0.8 and 2.5 years |
| **7.** | No impacted or ectopically erupted canines |
| **8.** | No extreme malocclusion (1 < overbite < 8 mm; 1 < overjet < 8 mm; 1 < crowding < 8 mm) |
| **9.** | No severe posterior crossbite at treatment start (> 3.5 mm total transversal discrepancy) |
| **10** | No active expansion of the dental arches (e.g. Hyrax or quad helix) |
| **11.** | No orthognathic surgery |
| **12.** | No primary disruption of treatment |

**Supplementary Table S2.** Differences considering the detected tooth movements between original and cleaned models, in each superimposition technique, performed with setting 1 at measurement point 1. One sample t-test shows if the mean difference is significantly different from zero in each case.

|  |  |  | | | | **95% Confidence Interval** | |
| --- | --- | --- | --- | --- | --- | --- | --- |
|  | **Original - Cleaned** | **t** | **df** | **P** | **Mean Difference** | **Lower** | **Upper** |
|  | A | 0.203 | 29 | 0.840 | 0.034 | -0.312 | 0.381 |
| **X** (mm) | B | 0.390 | 29 | 0.699 | 0.051 | -0.217 | 0.320 |
|  | C | -0.789 | 29 | 0.436 | -0.040 | -0.145 | 0.064 |
|  | A | -0.079 | 29 | 0.937 | -0.019 | -0.505 | 0.467 |
| **Y** (mm) | B | -0.198 | 29 | 0.844 | -0.028 | -0.317 | 0.261 |
|  | C | -0.564 | 29 | 0.577 | -0.016 | -0.072 | 0.041 |
|  | A | 0.615 | 29 | 0.544 | 0.106 | -0.247 | 0.459 |
| **Z** (mm) | B | 0.924 | 29 | 0.363 | 0.141 | -0.171 | 0.454 |
|  | C | 2.000 | 29 | 0.055 | 0.075 | -0.002 | 0.153 |
|  | A | -0.086 | 29 | 0.932 | -0.037 | -0.928 | 0.854 |
| **X – rot** (°) | B | 0.462 | 29 | 0.648 | 0.120 | -0.412 | 0.652 |
|  | C | 2.710 | 29 | 0.011^a^ | 0.356 | 0.087 | 0.624 |
|  | A | -1.671 | 29 | 0.105 | -0.771 | -1.714 | 0.172 |
| **Y – rot** (°) | B | -3.231 | 29 | 0.003^a^ | -1.215 | -1.984 | -0.446 |
|  | C | 0.255 | 29 | 0.800 | 0.027 | -0.186 | 0.239 |
|  | A | 0.237 | 29 | 0.814 | 0.203 | -1.545 | 1.951 |
| **Z – rot** (°) | B | -0.175 | 29 | 0.862 | -0.081 | -1.028 | 0.866 |
|  | C | 0.494 | 29 | 0.625 | 0.069 | -0.217 | 0.355 |

*p<0.002; Bonfrerroni correction applied

A, B, C correspond to the three superimposition techniques and reference areas tested in the study.

^a^These two cases approached, but did not reach the level of significance.

**Supplementary Table S3.** Mean of absolute differences of tooth movement measurements performed in original and cleaned models, with different settings, at the 1^st^ measurement period.

|  | | **Setting 1** | | | **Setting 2** | | |
| --- | --- | --- | --- | --- | --- | --- | --- |
|  | | **A** | **B** | **C** | **A** | **B** | **C** |
| **Linear measurements** | |  |  |  |  |  |  |
|  | X (mm) | 0.79 | 0.57 | 0.23 | 0.61 | 0.81 | 0.26 |
| **Incisor** | Y (mm) | 0.27 | 0.12 | 0.07 | 0.23 | 0.11 | 0.08 |
|  | Z (mm) | 0.41 | 0.48 | 0.18 | 0.53 | 0.54 | 0.21 |
|  | X (mm) | 0.33 | 0.34 | 0.09 | 0.61 | 0.49 | 0.10 |
| **Molar R** | Y (mm) | 1.06 | 0.66 | 0.11 | 1.00 | 0.76 | 0.12 |
|  | Z (mm) | 0.63 | 0.43 | 0.19 | 0.61 | 0.54 | 0.25 |
|  | X (mm) | 0.62 | 0.47 | 0.14 | 0.58 | 0.47 | 0.13 |
| **Molar L** | Y (mm) | 0.73 | 0.56 | 0.15 | 0.92 | 0.66 | 0.16 |
|  | Z (mm) | 0.92 | 0.62 | 0.13 | 0.66 | 0.66 | 0.14 |
|  | Median | 0.63 | 0.48 | 0.14 | 0.61 | 0.54 | 0.14 |
| **Angular measurements** | |  |  |  |  |  |  |
|  | X-rot (°) | 1.20 | 1.08 | 0.55 | 1.40 | 1.55 | 0.93 |
| **Incisor** | Y-rot (°) | 1.38 | 1.48 | 0.69 | 2.62 | 1.53 | 0.77 |
|  | Z-rot (°) | 2.12 | 1.40 | 0.56 | 3.09 | 2.34 | 0.90 |
|  | X-rot (°) | 0.83 | 0.81 | 0.47 | 2.80 | 1.34 | 0.54 |
| **Molar R** | Y-rot (°) | 1.54 | 1.41 | 0.32 | 1.85 | 1.55 | 0.37 |
|  | Z-rot (°) | 2.34 | 1.69 | 0.49 | 3.82 | 2.04 | 0.51 |
|  | X-rot (°) | 2.19 | 1.18 | 0.72 | 1.93 | 1.40 | 0.73 |
| **Molar L** | Y-rot (°) | 2.03 | 1.42 | 0.42 | 1.55 | 1.52 | 0.43 |
|  | Z-rot (°) | 3.05 | 1.81 | 0.65 | 1.82 | 2.11 | 0.61 |
|  | Median | 2.03 | 1.41 | 0.55 | 1.93 | 1.55 | 0.61 |

Friedman test showed significant differences between methods (p<0.001).

Pair-wise *a posteriori* tests (*Wilcoxon signed-rank test)* between measurements in original and cleaned models with each superimposition technique showed that superimposition techniques A and B did not differ significantly to each other, but both of them were different from technique C (p<0.01; Bonferroni correction applied). With both settings, there is a clear tendency that the smaller the reference area the higher the effect of artifacts in the detected tooth movement.

A, B, C correspond to the three superimposition techniques and reference areas tested in the study.

**Supplementary Table S4.** Mean of absolute differences of tooth movement measurements performed in the first versus the second scan of the cleaned models, with setting 1.

|  | | **Setting 1** | | |
| --- | --- | --- | --- | --- |
|  | | **A** | **B** | **C** |
| **Linear measurements** | |  |  |  |
|  | X (mm) | 0.34 | 0.21 | 0.07 |
| **Incisor** | Y (mm) | 0.08 | 0.11 | 0.07 |
|  | Z (mm) | 0.14 | 0.34 | 0.15 |
|  | X (mm) | 0.22 | 0.21 | 0.05 |
| **Molar R** | Y (mm) | 0.73 | 0.39 | 0.10 |
|  | Z (mm) | 0.60 | 0.21 | 0.15 |
|  | X (mm) | 0.28 | 0.23 | 0.08 |
| **Molar L** | Y (mm) | 0.50 | 0.30 | 0.17 |
|  | Z (mm) | 0.50 | 0.44 | 0.11 |
|  | Median | 0.43 | 0.23 | 0.10 |
| **Angular measurements** | |  |  |  |
|  | X-rot (°) | 0.98 | 1.40 | 0.85 |
| **Incisor** | Y-rot (°) | 1.19 | 0.87 | 0.85 |
|  | Z-rot (°) | 1.60 | 1.16 | 0.88 |
|  | X-rot (°) | 0.63 | 0.57 | 0.38 |
| **Molar R** | Y-rot (°) | 1.60 | 1.01 | 0.42 |
|  | Z-rot (°) | 1.48 | 0.83 | 0.66 |
|  | X-rot (°) | 0.83 | 1.16 | 0.87 |
| **Molar L** | Y-rot (°) | 1.78 | 0.90 | 0.25 |
|  | Z-rot (°) | 1.76 | 0.92 | 0.51 |
|  | Median | 1.48 | 0.92 | 0.66 |

Friedman test showed significant differences between methods (p<0.001).

Pair-wise *a posteriori* tests (*Wilcoxon signed-rank test)* between measurements in the first and the second scan of the cleaned models with each superimposition technique showed that superimposition techniques A and B did not differ significantly to each other, but both of them were different from technique C (p<0.01; Bonferroni correction applied). In all cases, there is a clear tendency that the smaller the reference area, the higher the effect of artifacts in the detected tooth movement.

A, B, C correspond to the three superimposition techniques and reference areas tested in the study.
